# Performance of Large Language Models in the Non-English Context: Qualitative Study of Models Trained on Different Languages in Chinese Medical Examinations

**DOI:** 10.2196/69485

**Published:** 2025-06-27

**Authors:** Zhong Yao, Liantan Duan, Shuo Xu, Lingyi Chi, Dongfang Sheng

**Affiliations:** 1 Department of Neurosurgery, Qilu Hospital of Shandong University, Jinan, China; 2 Cheeloo College of Medicine, Shandong University, Jinan, China; 3 Brain and Brain-Inspired Science, Shandong University, Jinan, China; 4 School of Management, Shandong University, No. 27 Shanda South Road，250100, Jinan, China, 86 15216408181

**Keywords:** large language model, medical examination, non-English, ChatGPT, language corpora

## Abstract

**Background:**

Research on large language models (LLMs) in the medical field has predominantly focused on models trained with English-language corpora, evaluating their performance within English-speaking contexts. The performances of models trained with non–English language corpora and their performance in non-English contexts remain underexplored.

**Objective:**

This study aimed to evaluate the performances of LLMs trained on different languages corpora by using the Chinese National Medical Licensing Examination (CNMLE) as a benchmark and constructed analogous questions.

**Methods:**

Under different prompt settings, we sequentially posed questions to 7 LLMs: 2 primarily trained on English-language corpora and 5 primarily on Chinese-language corpora. The models’ responses were compared against standard answers to calculate the accuracy rate of each model. Further subgroup analyses were conducted by categorizing the questions based on various criteria. We also collected error sets to explore patterns of mistakes across different models.

**Results:**

Under the zero-shot setting, 6 out of 7 models exceeded the passing level, with the highest accuracy rate achieved by the Chinese LLM Baichuan (86.67%), followed by ChatGPT (83.83%). In the constructed questions, all 7 models exceeded the passing threshold, with Baichuan maintaining the highest accuracy rate (87.00%). In few-shot learning, all models exceeded the passing threshold. Baichuan, ChatGLM, and ChatGPT retained the highest accuracy. While Llama showed marked improvement over previous tests, the relative performance rankings of other models stayed similar to previous results. In subgroup analyses, English models demonstrated comparable or superior performance to Chinese models on questions related to ethics and policy. All models except Llama generally had higher accuracy rates for simple questions than for complex ones. The error set of ChatGPT was similar to those of other Chinese models. Multimodel cross-verification outperformed single model, particularly improving accuracy rate on simple questions. The implementation of dual-model and tri-model verification achieved accuracy rates of 94.17% and 96.33% respectively.

**Conclusions:**

At the current level, LLMs trained primarily on English corpora and those trained mainly on Chinese corpora perform similarly well in CNMLE, with Chinese models still outperforming. The performance difference between ChatGPT and other Chinese LLMs are not solely due to communication barriers but are more likely influenced by disparities in the training data. By using a method of cross-verification with multiple LLMs, excellent performance can be achieved in medical examinations.

## Introduction

Large language models (LLMs) are advanced models trained on vast amounts of data, capable of processing natural language and performing specific tasks. In 2022, OpenAI released ChatGPT, which garnered widespread attention to LLMs. In the years that followed, numerous technology companies, including Meta with its Llama model and Google with PaLM 2, have also released their own LLMs. These models have been extensively researched and applied in various fields, including medicine. However, most existing research was conducted within English contexts, focusing on LLMs primarily trained on English-language corpora [1-3]. There is a notable lack of research on the performance of LLMs trained on non-English languages, particularly in non-English contexts.

To facilitate widespread adoption, LLMs should meet the requirements in diverse linguistic environments, not just within the English-speaking context. While many models offer multilingual versions, the training corpora may still be disproportionately weighted toward English, with insufficient representation of other languages, which could lead to subpar performance in non-English contexts [4]. Research has shown that ChatGPT, primarily trained on English-language corpora, is able to pass the United States Medical Licensing Examination [5], and can effectively addressing various clinical medical inquiries [6]. However, in some non-English medical examinations, ChatGPT fails to even reach a passing grade [7-9]. This disparity can be attributed to the preponderance of English-centric data in the training corpora, coupled with a limited inclusion of non-English content, leading to remarkable performance within English linguistic settings but less satisfactory results in non-English environments [10].

Researchers have made significant efforts to improve the performance of LLMs in non-English contexts. Wu et al [8] significantly enhanced ChatGPT’s performance in Chinese medical examinations by fine-tuning the model with external Chinese data, Zheng et al [11] developed a LLM named MOPH, trained on Chinese datasets, which outperformed ChatGPT in Chinese ophthalmology examinations. Considering the 3 major factors that influence their performance—computational power, algorithms, and training datasets—we hypothesize that making targeted adjustments to the training dataset can efficiently improve the performance of LLMs in non-English environments. In China, numerous LLMs have emerged, with training sets containing a greater proportion of Chinese-language data, theoretically leading to better performance in Chinese contexts. To assess this, we use the 2023 edition of Chinese National Medical Licensing Examination (CNMLE) as a benchmark to evaluate the performance of LLMs trained on different language corpora.

The primary objectives of this study are threefold: first, to conduct a batch test of Chinese LLMs; second, to compare the performance of Chinese and English LLMs; and third, to elucidate the commonalities and disparities in the performance characteristics of both types of models. The goal is to provide empirical evidence that can inform efforts to enhance the performance of LLMs in non-English contexts.

## Methods

The design and conduct of this study adhere to the METRICS (Model, Evaluation, Timing, Range/Randomization, Individual factors, Count, and Specificity of prompts and language) checklist.

### Selected LLMs and Test Duration

The models tested in this study include ChatGPT and Llama, which have undergone extensive research and are primarily trained on English-language corpora, as well as ERNIE Bot, Kimi, Baichuan, ChatGLM, and Yi-large, which have gained significant attention and are primarily trained on Chinese-language corpora. A detailed description of the tested models is provided in Table 1. To ensure consistency and reproducibility, all LLMs were tested with default parameters and in a zero-shot setting. In addition, to control for prompt engineering effects, tests were systematically replicated under a few-shot learning paradigm. The study was conducted from August to October 2024, and during this period, none of the tested models received version updates.

**Table 1.** Details of tested large language models.

Models	Version	Release year	Source code	Charge	Search internet capability	Source
ERNIE Bot	ERNIE-3.5‐8K	2023	Closed-source	Free	No	[12]
Kimi	moonshot-v1-8k	2023	Closed-source	Free	Yes	[13]
Baichuan	Baichuan3-Turbo	2023	Closed-source	Charge	Yes	[14]
ChatGLM	glm-4	2023	Open-source	Free	Yes	[15]
Yi-large	yi-large-turbo	2023	Closed-source	Charge	No	[16]
Llama	llama3-70b-instruct	2023	Open-source	Free	No	[17]
ChatGPT	GPT-4o	2022	Closed-source	Charge	No	[18]

### Resources and Transparency of Test Data

The test questions and standard answers for the 2023 CNMLE were obtained from publicly accessible networks, comprising a total sample of 600 multiple-choice questions. Each question stem was followed by multiple options, with only one correct answer. Participants were required to select the single correct answer from the provided options. A passing score was defined as achieving a correct answer rate higher than 60%.

### Self-Constructed Questions

Due to the real-time internet search capabilities of some tested LLMs, there is a possibility that these models could access standard answers from the internet or that their training datasets might already contain data from the CNMLE, leading to data contamination. To rule out these scenarios, a panel of three experienced physicians constructed a set of 100 novel questions that closely mirror the format and style of the CNMLE. These 100 questions are not retrievable from the internet and are guaranteed not to be present in the training datasets of the LLMs. The self-constructed questions were presented to the LLMs using the same prompts, and the accuracy rate was calculated. The consistency in the accuracy rates of the LLMs when answering both the self-constructed questions and the CNMLE questions was compared to assess whether the LLMs’ performance relied on real-time web retrieval or the presence of data contamination. The responses from the LLMs were generated under standard conditions, with detailed answers and analysis provided in Multimedia Appendix 1.

### Range and Randomization of the Topics Tested

The test data comprise all questions from the most recent 2023 edition of CNMLE without artificial selection. The examination is structured into 4 sections: Basic Medical Sciences, Medical Humanities, Clinical Medicine, and Preventive Medicine, covering various fields and directions in medicine [19]. Given that an average of over half a million medical students participate in the CNMLE examination annually, the compilation of its questions follows rigorous and standardized criteria, establishing it as a highly representative assessment of medical knowledge.

### Specificity of the Prompt and Language

First, we implemented a zero-shot configuration to ensure experimental reproducibility. Then, to control for potential confounding effects of prompt engineering, we conducted additional validation using few-shot learning protocols, which was conducted with 3 demonstration examples per task based on the standardized protocol. Using the prompt “You are a doctor. I will give you a multiple-choice question from a medical exam. Please read the question carefully, choose the most appropriate answer from the five options, and explain the reason,” we called the application programming interface to sequentially present 600 questions to the LLMs and obtained their answers and explanations. The language for the prompts, inputs, and outputs was Chinese. Chinese includes Mandarin and minority languages, but this study specifically refers to Mandarin. To eliminate any potential impact of language barriers on model performance, we conduct a professional-grade translation via DeepL, followed by cross-verification by a bilingual scholar to eliminate significant semantic distortions, and then followed the same procedure to ask the questions to English-language LLMs.

### Evaluation of LLM Responses

The accuracy of the answers provided by the LLMs was determined by comparing them to the officially published correct answers. The evaluation process was entirely objective, with no individual subjective involvement. Each answer, along with its corresponding explanation, was thoroughly examined to identify the underlying reasons for any errors. For each model, a set of incorrectly answered questions was compiled to create an error set, and a Venn diagram was generated to analyze the overlap and correlations between the error sets [20].

### Subgroup Analysis

The test questions vary in type, content breadth, and difficulty level, which may affect the performance of LLMs. We conducted a subgroup analysis of the questions to rule out potential confounding factors and also to assess whether the performance of these models across various subgroups conforms to general logical cognition.

Differences in policies and ethics among countries, such as those related to euthanasia and abortion, may influence the responses given by LLMs. Based on the knowledge scope, the questions were classified into 2 categories: policies and ethics, and professional knowledge.

Questions were also differentiated based on their polarity; those requiring a correct or affirmative answer were termed “positive questions,” while those necessitating the identification of an incorrect or negative answer were termed “negative questions,” with the latter generally recognized as more challenging [21].

Furthermore, questions were categorized by their formats into medical knowledge, which tests a single knowledge point directly, and clinical case analysis, which involves specific clinical scenarios that require the integration of information to formulate a response, thereby increasing the difficulty. According to CNMLE statistics, the correct answer rate for clinical case analysis is lower than that for medical knowledge.

In alignment with Bloom’s Taxonomy, an educational classification system for cognitive skills, the required cognitive abilities are hierarchically ranked from low to high as remembering, understanding, applying, analyzing, evaluating, and creating. The first 3 are defined as lower-order thinking, while the latter 3 are considered higher-order thinking [22].

Based on the above grouping criteria, each question was assigned to a subgroup after consensus was reached through discussion among 3 doctors.

### Ethical Considerations

This study did not involve any human participants, thereby waiving the need for institutional review board review.

## Results

### Accuracy Rate

**Figure 1.**
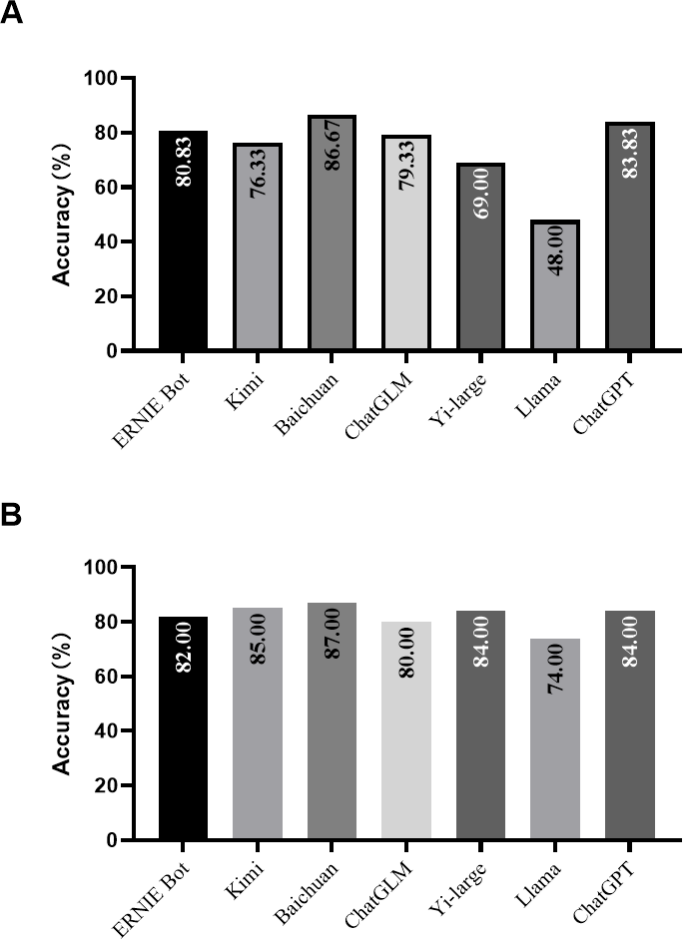
The accuracy rates of seven large language models in the Chinese National Medical Licensing Examination (**A**) and self-constructed questions (**B**).

Figure 1A illustrated the accuracy rates of 7 LLMs in the CNMLE, ranked from highest to lowest: Baichuan (86.67%), ChatGPT (83.83%), ERNIE Bot (80.83%), ChatGLM (79.33%), Kimi (76.33%), Yi-large (69.00%), and Llama (48.00%). All models, with the exception of Llama, surpassed the passing threshold, with the Chinese model Baichuan emerging as the top performer, outscoring even ChatGPT. In a set of 100 self-constructed questions, Llama’s accuracy rate saw a significant improvement, rising from 48% to 74%, whereas the other models’ performance remained largely consistent with their results in the CNMLE. Baichuan maintained the highest accuracy at 87.00%, followed by Kimi at 85.00% and ChatGPT at 84.00% (Figure 1B). When the questions were translated into English and represented to ChatGPT using the same method, its accuracy rate decreased to 81.00%, likely due to errors introduced during the translation process. We re-evaluated the models using the few-shot approach, and observed that the relative rankings among the models remained largely consistent. Baichuan (84.50%) demonstrated the highest accuracy rate, followed by ChatGLM (84.50%) and ChatGPT (81.83%). Notably, Llama exhibited a significant improvement in accuracy rate after prompt engineering modifications (Multimedia Appendix 1). Overall, the results of the second test exhibited trends comparable to existing findings, indicating that the prompt engineering has minimal influence on the relative performance among models.

### Subgroup Analyses

To analyze the performance of LLMs across various question types, the questions were categorized based on distinct characteristics. The categorization was as follows: by knowledge scope, questions were divided into policies and ethics (28 questions) and professional knowledge (572 questions); by question polarity, into positive questions (541 questions) and negative questions (59 questions); by question format, into medical knowledge (245 questions) and clinical case analysis (355 questions); and by Bloom’s Taxonomy, into lower-order thinking (298 questions) and higher-order thinking (302 questions).

**Figure 2.**
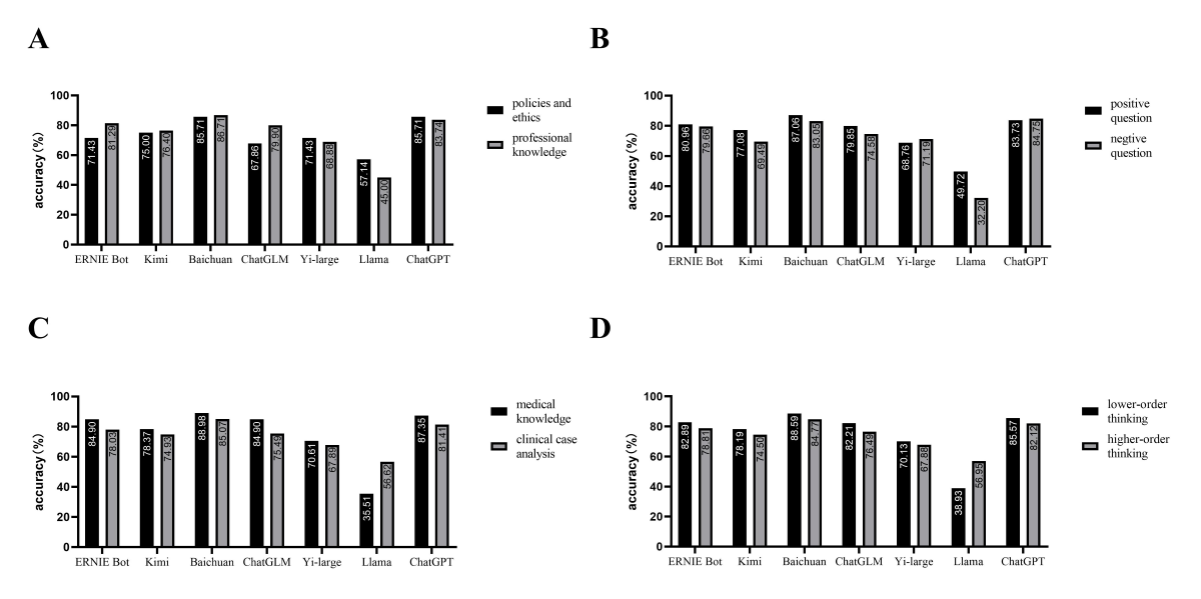
The accuracy rates of 7 large language models in the Chinese National Medical Licensing Examination questions stratified into subgroups according to different criteria: knowledge scope (**A**), question polarity (**B**), question format (**C**), and Bloom’s taxonomy (**D**).

Figure 2A illustrated that, among the policies and ethics questions, ChatGPT achieved the highest accuracy rate (85.71%), while Baichuan performed best on professional knowledge questions with an accuracy rate of 86.71%. Interestingly, 4 Chinese-language models (ERNIE Bot, Kimi, Baichuan, and ChatGLM) demonstrated lower accuracy on policies and ethics questions than on professional knowledge questions. In contrast, the 2 English-language models (Llama and ChatGPT) exhibited higher accuracy on policies and ethics questions compared to professional knowledge questions. This suggests that, despite the differences in policies and ethical concerns across countries, English-language models may account for these variations, enabling them to perform better on policies and ethics questions in Chinese.

As depicted in Figure 2B, Baichuan (87.06%) and ChatGPT (84.75%) achieved the highest accuracy rates for positive and negative questions, respectively. Five models (ERNIE Bot, Kimi, Baichuan, ChatGLM, and Llama) exhibited higher accuracy on positive questions than on negative ones, while ChatGPT and Yi-large showed a modestly better performance on negative questions.

Figure 2C showed that 6 models (ERNIE Bot, Kimi, Baichuan, ChatGLM, Yi-large, and ChatGPT) had higher accuracy rates for medical knowledge questions than for clinical case analysis questions. Conversely, Llama’s performance was superior on clinical case analysis questions compared to medical knowledge questions.

A similar trend is observed in Figure 2D, where 6 LLMs (ERNIE Bot, Kimi, Baichuan, ChatGLM, Yi-large, and ChatGPT) achieved better accuracy on lower-order thinking questions than on higher-order thinking questions. Llama, however, exhibited the opposite trend, with a higher accuracy rate on higher-order thinking questions than on lower-order ones. The greater frequency of errors on simple questions compared to complex ones by Llama suggests that its atypical performance may stem from difficulties in accurately understanding and processing the Chinese language, which contributed to its overall lower accuracy. ChatGPT’s performance across the subgroups was consistent with that of multiple Chinese language models, aligning with expectations based on the volume of Chinese-language data available to the models, with performance differences likely reflecting variations in data quantity.

### Error Sets

**Figure 3.**
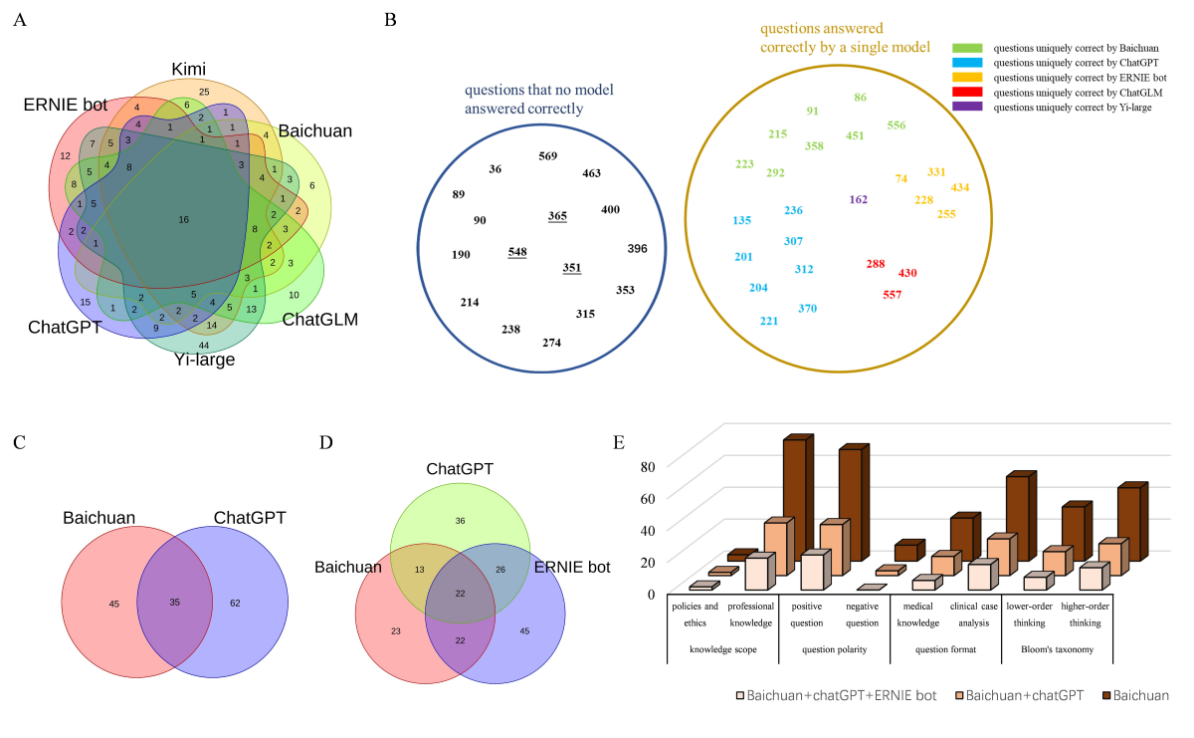
Collect the incorrectly answered questions for each model to create error sets, and Venn diagram demonstrated the intersection of the error set of 6 models (**A**); incorrectly answered questions (**B**); intersection of the error sets of the top two (**C**) and the top three models (**D**) with the highest accuracy; column chart exhibits the comparison of the number of incorrect answers between single model and multiple model cross-verification (**E**).

As shown above, with the exception of Llama, the overall performance and trends in subgroup performance of the 6 other LLMs were largely consistent. We collected the error sets of these 6 models, identified their intersections, and visualized them using a Venn diagram. Figure 3A demonstrates that there were 16 common incorrect questions that all LLMs answered incorrectly. Within ChatGPT’s error set, there were 15 unique incorrect questions that were answered incorrectly by ChatGPT alone, despite being correctly addressed by the other models. Similarly, the error sets of ERNIE Bot, Kimi, Baichuan, ChatGLM, and Yi-large contained unique incorrect questions, with 12, 25, 6, 10, and 44 such instances, respectively.

We conducted enhanced error profile analysis by aggregating incorrectly answered questions. The error set revealed 16 questions consistently misanswered by all 6 models, with 13 questions demonstrating divergent error patterns across different LLMs, while 3 questions exhibited identical incorrect selections across all 6 models. Besides, 25 questions were uniquely answered correctly by individual models, with specific distributions: 8 by Baichuan, 8 by ChatGPT, and 5 by ERNIE-Bot. As in Figure 3B, the blue circle demonstrates questions that no model answered correctly and yellow circle shows those answered correctly by only one model, the numbers within the circles indicate the question identifiers and numerical values in distinct colors represent questions that were exclusively answered correctly by a single LLM.

Considering the intersection of the error sets between the 2 models with the highest accuracy rates, ChatGPT and Baichuan, there are 35 common incorrect questions. Expanding to the three most accurate models, Baichuan, ChatGPT, and ERNIE Bot, their intersection contained only 22 common incorrect questions (Figures 3C and 3D).

Comparative analysis between single-model (Baichuan) and multimodel (Baichuan+ChatGPT, Baichuan+ChatGPT+ERNIE bot) approaches demonstrated significant hallucination rate reduction through cross-verification. The most pronounced improvements occurred in professional knowledge and lower-order thinking domains, suggesting that cross-verification mechanisms primarily mitigate artificial intelligence (AI) hallucination in “simple” question types (Figure 3E). Previous studies have suggested that using multiple LLMs for cross-verification can effectively mitigate AI hallucination [23,24]. If we simultaneously use 2 models (Baichuan+ ChatGPT) to take a medical examination, adopting the LLMs’ answer when they agree and resorting to human judgment when they disagree, we could potentially achieve an accuracy rate of 94.17%. Moreover, using the same approach with three models (Baichuan+ChatGPT+ERNIE bot) could further improve accuracy to 96.33%.

## Discussion

### Principal Findings

Research on LLMs in English contexts has been extensive and thorough, whereas studies in non-English contexts remain relatively underexplored. Beyond ChatGPT, which is trained primarily on English corpora, LLMs developed using non-English language datasets also hold significant value for research and testing. Such studies could provide valuable insights for optimizing ChatGPT or developing new LLMs tailored specifically for non-English environments. We take a model testing approach by comparing LLMs mainly trained on English corpora with LLMs trained on Chinese corpora, analyzing the impact of variations in training data composition or domain-specific weighting on LLMs’ performance.

This study used the CNMLE as a testing tool, which may not fully reflect the performance of LLMs in real-world medical scenarios. Nevertheless, we argue that if an LLM struggles to perform well in medical examinations, it is even less likely to succeed in practical medical applications. We selected medical examination questions as the testing tool for LLMs for 2 main reasons. First, these questions are highly representative: the CNMLE serves as the qualifying examination for Chinese medical students, covering a wide-ranging knowledge base and various question types. Annually, more than 500,000 students take the examination, and the stable pass rate over the years suggests that the difficulty level remains consistent, making the CNMLE a reliable and representative benchmark for testing. Second, the examination questions are relatively standardized. In this testing, we used a zero-shot setting to ensure the reproducibility of experimental results. In the field of natural language processing, standardized test sets are commonly used to evaluate LLMs, ensuring the comparability and reproducibility of research findings [25,26]. It is recommended that future assessments of LLMs in the medical domain also incorporate standardized test sets, in spite of the complexities of real-world medical scenarios.

The findings of this study indicate that the English LLM ChatGPT and several Chinese LLMs perform comparably in a Chinese context. Despite being a leading model among LLMs, ChatGPT does not exhibit a significant advantage in Chinese contexts. While we initially speculated that language communication barriers might contribute to this outcome, our study and previous research have demonstrated no significant difference in LLM performance when questions are translated into English versus being asked directly in Chinese [27,28]. This suggests that the performance gap in Chinese contexts is not primarily due to communication barriers but is more likely attributable to the limited representation of Chinese corpora in the training datasets. The disparity between Chinese and English datasets goes beyond natural language processing challenges and includes differences in content, such as epidemiology, traditional Chinese medicine, policy, and ethics. Since English LLMs are predominantly trained on English-language datasets, their ability to understand and respond effectively in non–English language contexts, as in Chinese medical examinations, is constrained. Notably, Llama3 performed poorly in this study, which may be attributed to its limited proficiency in Chinese or the scarcity of medical-specific training data. In contrast, models like PMC-Llama, fine-tuned on the Llama platform specifically for medical applications, have demonstrated excellent performance in other studies [29].

Previous studies have assessed ChatGPT’s performance in non-English medical examinations and explored ways to enhance its capabilities, such as leveraging external data, developing task-specific models, and using cross-lingual models like XLM. XLM leverages accurate language translation to use English data for solving non-English problems, which is a viable solution when non-English training datasets are limited. Our findings indicate that some LLMs trained on Chinese-language corpora have generally matched or surpassed the performance of ChatGPT in the CNMLE, even with shorter training times and less data. This prompts us to reconsider whether the pursuit of an exhaustive training dataset, encompassing all languages and cultures, is necessary, or if developing cost-effective, field-specific LLMs might be more prudent. Among the 7 LLMs tested, 6 exceeded the passing level, and 5 surpassed Med3R, a model specifically developed for Chinese medical examinations. Med3R, released in 2018, achieved a 76% accuracy rate in the 2017 edition of CNMLE [30]. Yet, within a few years, most tested LLMs have exceeded Med3R’s performance, demonstrating the superiority of these general-purpose LLMs over traditional models, despite not being specifically designed for medical testing. Notably, ChatGPT showed substantial improvement in CNMLE compared to its earlier versions, underscoring the continual evolution of LLMs as a key advantage [28,31]. However, as the training dataset expands, the associated costs increase rapidly, and when marginal effects are observed, the cost-benefit ratio must be considered. In LLMs, diminishing marginal returns refer to the phenomenon where, as the model size increases, the performance gains from adding the same amount of parameters or computational resources gradually decrease. This is a crucial aspect of scaling law and has significant implications for understanding and decision-making in model design and deployment strategies.

While this study exclusively used Chinese for evaluation, its findings demonstrate generalizable implications for other non–English language environments. Given that LLMs predominantly derive training data from web-based textual corpora—where English constitutes the most prevalent linguistic resource (53.8% of indexed content) followed by Chinese (14.3%) according to the Ethnologue linguistic database—Chinese is classified as a high-resource language [32]. Notably, despite ChatGPT’s training corpus incorporating Chinese linguistic data, its CNMLE performance underperformed compared to Baichuan, which was specifically optimized with Chinese medical corpora. This disparity suggests that in low-resource language contexts, the performance gap between generalist LLMs like ChatGPT and linguistically specialized models may prove even more pronounced. For LLMs to achieve broader applicability and widespread deployment in the future, their adaptability to non-English and low-resource language contexts must be prioritized. Further research is needed to identify the most effective and cost-efficient approaches for improving model performance in these settings.

LLMs are poised to enable profound and transformative integration within health care systems, necessitating systematic incorporation into clinical workflows by medical practitioners. Some scholars even propose that prompt engineering may become a core component of medical education in the future [33]. In addition, it is essential to identify and address potential challenges during the deployment of LLMs in health care settings. These insights will guide the ongoing optimization of these models and help mitigate the risks associated with their inappropriate application. Given that real-world clinical scenarios are more complex than machine-simulated environments, continuous, real-world application and rigorous testing are essential for steering the iterative improvements of LLMs. To become effective tools in the medical field, these models must undergo a repeated cycle of testing, optimization, retesting, and refinement, progressively advancing to meet the rigorous demands of clinical reliability and usability. We hope that our research will offer valuable insights into the optimization of LLMs, particularly in non-English linguistic environments.

### Limitations

This study exclusively used standardized test questions, potentially limiting its generalizability to real-world medical applications. We plan to extend this work by integrating open-ended clinical problem-solving tasks to assess LLM performance under practical conditions. In addition, our cross-verification process remained semiautomated, requiring human judgment when models disagree. Subsequent efforts will prioritize developing fully automated cross-verification protocols.

### Conclusions

At the current stage, LLMs trained primarily on English corpora and those trained mainly on Chinese corpora exhibited comparable performance in CNMLE, with models trained on Chinese corpora maintaining a slight advantage. Subgroup analyses and error set evaluations indicate similar performance between ChatGPT and various Chinese LLMs. The performance differences between Chinese- and English-trained models are not solely attributed to communication barriers but are more likely influenced by disparities in the training data. Using a cross-verification approach across multiple LLMs can achieve exceptional results in CNMLE.

## Supplementary material

10.2196/69485 Multimedia Appendix 1 Additional material.

## Abbreviations

AI: artificial intelligence
CNMLE: Chinese National Medical Licensing Examination
LLM: large language model
METRICS: Model, Evaluation, Timing, Range/Randomization, Individual factors, Count, and Specificity of prompts and language
